# Supplementary material for: Rapid detection and molecular epidemiology of β-lactamase producing Enterobacteriaceae isolated from food animals and in-contact humans in Nigeria
Source: PLoS One. 2024 Apr 11;19(4):e0289190. doi: 10.1371/journal.pone.0289190 (PMC11008865; doi:10.1371/journal.pone.0289190)
Supplement: S1 Table — (DOCX) [file pone.0289190.s001.docx]

**Rapid detection and molecular epidemiology of β-lactamase producing *Enterobacteriaceae* isolated from food animals and in-contact humans in Nigeria.**

Solomon O. Olorunleke, M. Kirchner, N. Duggett, M. K. Stevens, K. F. Chah, J.A Nwanta, L. A. Brunton, and M. F. Anjum.

**S1 Table. AMR genotype of each isolate in the control panel**

| Strain ID | Organism | AMR genotype whole-genome sequence | β-Lactamase genes detected by multiplex RT-PCR |
| --- | --- | --- | --- |
| HPA 70 | *Klebsiella pneumonia* | aac3, aac6, aadA6, strA, strB, BIL-1, CMY-6, **CTX-M-15**, LAT-1, **NDM-1**, OHIO-1, OXA-1, **SHV-67**, **TEM-1b**, catA1, oqxA6, oqxA7, oqxB19, qnrS1, sul1 | 16S, TEM, SHV, CTX-M |
| HPA 5 | *Acinetobacter baumanni* | aac2, aac3, aadA1, ant3, strA, strB, ADC-1, OXA-58, OXA-83, PER-1, catA1, sul1, sul2 |  |
| HPA 77 | *Klebsiella pneumonia* | strA, OHIO-1, **OXA-48**, **SHV-40**, OXA-405, **TEM-1b**, catA1, fosA, oqxA10, oqxb20, sul1, tet-C, dfrA5 | 16S, TEM, SHV |
| B2304 | *Escherichia coli* | aac6, aadA2, ant3, strA, strB, **TEM-1b**, cml, sul3, tet-AB, dfrA12 | 16S, TEM |
| HPA 59 | *Klebsiella pneumonia* | aac6, aadA6, ant3, strA, strB, aac3, **CTX-M-15**, OHIO-1, OXA-1, OXA-9, **SHV-11**, **TEM-1b**, catA1, cml, oqxA6, oqxB20, arr-2, sat2A, sul1, sul2, dfrA1 | 16S, TEM, SHV, CTX-M |
| HPA 7 | *Acinetobacter baumanni* | aac2, aac3, strA, strB, ADC-1, OXA-24, OXA-79, sul1 |  |
| HPA 47 | *Klebsiella pneumonia* | strA, OHIO-1, **OXA-48**, **SHV-40**, OXA-405, **TEM-1b**, catA1, oqxA10, oqxB20, sul1, tet-C, dfrA14 | 16S, TEM, SHV |
| HPA 26 | *Enterobacter spp* | aac3, aac6, ant3, strA, strB, BIL-1, CMY-6, **CTX-M-15**, LAT-1, **NDM-1**, OXA-1, ACT-25, **TEM-1b**, qnrB1, sul1, sul2, tet-D, dfrA14 | 16S, TEM, CTX-M |
| HPA 46 | *Klebsiella pneumonia* | aadA6, aac6, **CTX-M-15**, OHIO-1, OXA-1, **OXA-48**, **SHV-11**, OXA-405, **TEM-1b**, oqxA6, oqxB19, tet-A | 16S, TEM, SHV, CTX-M |
| HPA 29 | *Escherichia coli* | aac6, aadA2, strA, **KPC-3**, OHIO-1, OXA-9, **SHV-158**, **TEM-1**, catA1, mphA, oqxA1, oqxB2, sul1, dfrA12 | 16S, TEM, SHV |
| HPA 37 | *Escherichia coli* | aac3, aac6, aadA2, ant3, strA, strB, BIL-1, CMY-4, **CTX-M-15**, LAT-1, **NDM-1**, OXA-1, **TEM-1b**, catA1, cml_A1, mphA, sul1, tet-A, dfrA12 | 16S, TEM, CTX-M |
| B2319 | *Salmonella spp* | aac6, **CTX-M-15**, **TEM-1b** | 16S, TEM, CTX-M |
| HPA 31 | *Escherichia coli* | **CTX-M-26**, OHIO-1, **SHV-40**, **TEM-1b**, oqxA6, oqxB25 | 16S, TEM, SHV, CTX-M |
| B2320 | *Salmonella spp* | aac6, **TEM-1b** | 16S, TEM |
| B2318 | *Escherichia coli* | aadA1, ant3, OXA-1, catA1, sul1, tet-AB | 16S |
| B2308 | *Escherichia coli* | aac6, aadA2, ant3, OHIO-1, OXA-9, **SHV-12**, **TEM-1**, catA1, mphA, sat2, sul1, tet-AB, dfrA12 | 16S, TEM, SHV |
| B2322 | *Klebsiella* | OHIO-1, **SHV-40**, **TEM-1b**, catA1, oqxA6, oqxB25, sul1, dfrA5 | 16S, TEM, SHV |
| HPA 93 | *Pseudomonas aeroginosa* | aac6, aadA1, ant2, ant3, strA, OXA-10, **VIM-2**, VEB-9, catB7, sul1, dfrB2 |  |
| B2326 | *Salmonella spp* | aac6, ant3, **CTX-M-2**, **TEM-1b**, sul1, tet-A, dfrA1 | 16S, TEM, CTX-M |
| HPA 96 | *Psuedomonas fluoresens/putida* | aac6, ant2, **VIM-2**, sul1, dfrA1 |  |
| HPA 76 | *Klebsiella pneumonia* | aac6, aadA6, OHIO-1, **SHV-12**, **VIM-1**, oqxA5, oqxB18, qnrS1, dfrA14 | 16S, SHV |
| NCTC13438 | *Escherichia coli* | aac6, aadA2, strA, **KPC-3**, OHIO-1, OXA-9, **SHV-158**, **TEM-1**, catA1, mphA, oqxA1, oqxB2, sul1, dfrA12 | 16S, TEM, SHV |
