# Supplementary material for: Rapid detection and molecular epidemiology of β-lactamase producing Enterobacteriaceae isolated from food animals and in-contact humans in Nigeria
Source: PLoS One. 2024 Apr 11;19(4):e0289190. doi: 10.1371/journal.pone.0289190 (PMC11008865; doi:10.1371/journal.pone.0289190)
Supplement: S3 Table — (DOCX) [file pone.0289190.s003.docx]

**Rapid detection and molecular epidemiology of β-lactamase producing *Enterobacteriaceae* isolated from food animals and in-contact humans in Nigeria.**

Solomon O. Olorunleke, M. Kirchner, N. Duggett, M. K. Stevens, K. F. Chah, J.A Nwanta, L. A. Brunton, and M. F. Anjum.

S3 Table. Primer and Probe Sequence

| Gene | Primer Sequence 5’ – 3’ | Reference |
| --- | --- | --- |
| TEM | Forward GCATCTTACGGATGGCATGA  Reverse GTCCTCCGATCGTTGTCAGAA  Probe [6FAM] CAGTGCTGCCATAACCATGAGTGA [BHQ1] | (Roschanski et al., 2014) |
| SHV | Forward TCCCATGATGAGCACCTTTAAA  Reverse TCCTGCTGGCGATAGTGGAT  Probe [Cyanine5] TGCCGGTGACGAACAGCTGGAG [BHQ2] |  |
| CTX-M | Forward ACCGAGCCSACGCTCAA  Reverse CCGCTGCCGGTTTTATC  Probe [Cyanine3] CCCGCGYGATACCACCACGC [BHQ2] |  |
| 16S-RNA | Forward CCTCTTGCCATCGGATGTG  Reverse GGCTGGTCATCCTCTCAGACC  Probe [TexasRed] GTGGGGTAACGGCTÇACCTAGGCGAC [BHQ2] | APHA |

**Reference**

Roschanski N, Fischer J, Guerra B, Roesler U. Development of a Multiplex Real-Time PCR for the Rapid Detection of the Predominant Beta-Lactamase Genes CTX-M, SHV, TEM and CIT-Type AmpCs in Enterobacteriaceae. Bereswill S, editor. PLoS One. 2014;9: e100956. doi:10.1371/journal.pone.0100956
