# Supplementary material for: Rapid detection and molecular epidemiology of β-lactamase producing Enterobacteriaceae isolated from food animals and in-contact humans in Nigeria
Source: PLoS One. 2024 Apr 11;19(4):e0289190. doi: 10.1371/journal.pone.0289190 (PMC11008865; doi:10.1371/journal.pone.0289190)
Supplement: S4 Table — (DOCX) [file pone.0289190.s004.docx]

**Rapid detection and molecular epidemiology of β-lactamase producing *Enterobacteriaceae* isolated from food animals and in-contact humans in Nigeria.**

Solomon O. Olorunleke, M. Kirchner, N. Duggett, M. K. Stevens, K. F. Chah, J.A Nwanta, L. A. Brunton, and M. F. Anjum.

S4 Table. β-Lactamase RT- PCR Reaction mixture

| RT-PCR Mixture | Volume |
| --- | --- |
| Qiagen 2x multiplex master mix (QuantiTect Multiplex PCR) | 12.5 µl |
| Multiplex primer equimolar mix (CTX, TEM, SHV- 1µl each of forward and reverse primers at conc. 10pmol) | 6 µl |
| 0.4 µl each of 16S forward and reverse primer (conc. 10µM) | 0.8 µl |
| 0.1 µl of TEM Tag Man probe (conc. 5pmol) | 0.1 µl |
| 0.2 µl of each of CTX and TEM probes (conc. 5pmol) | 0.4 µl |
| 0.2 µl of 16S probe (conc. 1 µM) | 0.4 µl |
| RNase free water | 4 µl |
| DNA template | 1 µl |
| Total volume for reaction | **25 µl** |
