# Supplementary material for: Rapid detection and molecular epidemiology of β-lactamase producing Enterobacteriaceae isolated from food animals and in-contact humans in Nigeria
Source: PLoS One. 2024 Apr 11;19(4):e0289190. doi: 10.1371/journal.pone.0289190 (PMC11008865; doi:10.1371/journal.pone.0289190)
Supplement: S5 Table — (DOCX) [file pone.0289190.s005.docx]

**Rapid detection and molecular epidemiology of β-lactamase producing *Enterobacteriaceae* isolated from food animals and in-contact humans in Nigeria.**

Solomon O. Olorunleke, M. Kirchner, N. Duggett, M. K. Stevens, K. F. Chah, J.A Nwanta, L. A. Brunton, and M. F. Anjum.

S5 Table. RT-PCR conditions for 16S, TEM, SHV, and CTX-M Reaction

| Cycling Temperature | Time | Number of Cycles |
| --- | --- | --- |
| 95^ᵒ^C | 15 min | Preliminary heating |
| 95^ᵒ^C | 30 sec | **30 Cycles** |
| 50^ᵒ^C | 90 sec |  |
| 70^ᵒ^C | 60 sec |  |
