# Supplementary material for: Rapid detection and molecular epidemiology of β-lactamase producing Enterobacteriaceae isolated from food animals and in-contact humans in Nigeria
Source: PLoS One. 2024 Apr 11;19(4):e0289190. doi: 10.1371/journal.pone.0289190 (PMC11008865; doi:10.1371/journal.pone.0289190)
Supplement: Attachment — Submitted filename: P-ONE-review-08-10-23.docx [file pone.0289190.s008.docx]

**Title: Rapid detection and molecular epidemiology of β-lactamase producing Enterobacteriaceae isolated from food animals and in-contact humans in Nigeria.**

Authors: Olorunleke et al.

**General Comments**

The article is well written and seems to be the first to conduct in-depth studies on Enterobacteriaceae in three states in the South East of Nigeria. The report of pan resistance in some of these states is quite alarming also. However, there are a few grey areas that need to be explained.

**Specific comments**

Is it possible to add the cost of multiplex RT-PCR as against the cost of culture in our African setting where qPCR although on the increase in terms of usage due to covid, might not be popular for AMR?

In the supplementary Tables 2 & 6, genus and species names were not italicized.

I was looking out for the common genes that are shared in the states and which ones are found in the three states. In addition, I was looking forward to see at a glance the human isolates according to states and sites and the genes that are similar in each state since human isolates are 34 in number. This table should show any possible similarity with the animal species. I mean the AMR Table not plasmid table. I know there are comprehensive tables as supplementary files, but I would like to see at a glance a table with genes that are common and cut across the states.

Page 22 line 420, has an incomplete sentence, Lines 421 in page 22 to 423 in page 23, needs to be re-casted.

Lines 387 – 390, authors attributed highest prevalence of blaTEM 388 and blaSHV in Abia to be due to its commercial hub, while this may be partly true, it can also be seen from their results that Abia state had the lowest number of samples and so this could also be another attributable factor.
