# Supplementary material for: Rapid detection and molecular epidemiology of β-lactamase producing Enterobacteriaceae isolated from food animals and in-contact humans in Nigeria
Source: PLoS One. 2024 Apr 11;19(4):e0289190. doi: 10.1371/journal.pone.0289190 (PMC11008865; doi:10.1371/journal.pone.0289190)
Supplement: Attachment — Submitted filename: Response to Reviewers_PLOS ONE_SO.docx [file pone.0289190.s009.docx]

Dr Solomon O. Olorunleke

Department of Animal Science

Ebonyi State University, Abakaliki

Nigeria

11/12/2023

Kwame Kumi Asare, PhD

Academic Editor

PLOS ONE

Dear Dr Asare,

**Re: PONE-D-23-21745, Rapid detection and molecular epidemiology of β-lactamase producing Enterobacteriaceae isolated from food animals and in-contact humans in Nigeria**

I hope this letter finds you well. I am writing in response to the invaluable feedback provided by the four esteemed academic reviewers regarding our manuscript titled " Rapid detection and molecular epidemiology of β-lactamase producing Enterobacteriaceae isolated from food animals and in-contact humans in Nigeria." We appreciate the time and effort invested by the reviewers in critically evaluating our work, and we are grateful for their constructive comments.

Reviewer 1:

We sincerely appreciate your positive feedback and constructive comments on our manuscript, your encouraging words are both motivating and affirming, and we are grateful for the recognition of the potential impact of our study in resource-constrained environments. We are particularly pleased that you found the manuscript to be well-written and described. Your acknowledgement of the potential contribution to knowledge in low-resource settings is a testament to our commitment to addressing challenges faced in such environments. We share your vision that the methods outlined in our study can be instrumental in establishing efficient surveillance practices with reduced turnaround time.

Reviewer 2:

We would like to express our sincere gratitude for your thoughtful and comprehensive feedback on our manuscript. Your detailed comments and insights have been instrumental in refining our work, and we highly appreciate the time and effort you invested in the review process.

Addressing your concerns and suggestions, we have implemented the following revisions:

- Acronym Usage (RT-PCR): We appreciate your clarification regarding the use of "RT-PCR." We have revised the manuscript to replace it with "qPCR" to accurately reflect our methodology.
- Focus on ESKAPE Pathogens: We acknowledge your point about the focus on Enterobacteriaceae rather than all ESKAPE pathogens. We have adjusted the conclusion and recommendations to better align with the main study findings, ensuring a more cohesive narrative.
- Sensitivity and Specificity Interpretation: Sensitivity and specificity validation for the qPCR primers and probes was conducted using control strains rather than test isolates. Contrary to the impression given, the number of negative controls exceeded four, and the selection of control strains was meticulous. These strains served as both positive and negative controls for the detection of various ESBL genes, namely CTX-M (18 positives and 4 negatives), TEM (16 positives and 6 negatives), SHV (11 positives and 11 negatives), and 16s (18 positives and 4 negatives). It is crucial to clarify that the four isolates utilized as negative controls in the test isolate experiment underwent validation only after confirming the sensitivity and specificity of the primers and probes for detecting ESBLs.
- Justification for ESBL Genes Focus: We have included a brief justification for focusing on the three ESBL genes in the introduction (Lines 70 – 82), supported by relevant references to provide context for our selection.
- Figure 1 and Table 1 Corrections: We have corrected the discrepancies in Figure 1 and addressed your suggestion to streamline Table 1 by providing a concise statement indicating 100% sensitivity, specificity, accuracy, and precision.
- Inclusion of Study Limitations: We have added a section specifically addressing the limitations of our study, including the limited sample size and the restricted number of Klebsiella and Enterobacter strains tested.
- Repetitive Sections and Unwieldy Phrases: We have revised and rephrased sections that were identified as repetitive or unwieldy, following your suggestions.
- Minor Revisions: We have addressed the minor revisions you pointed out, including clarifications on terms, grammar improvements, and adjustments to the discussion section.

Reviewer 3:

We acknowledge the concerns raised by Reviewer 3 regarding the evaluation of the assay's rapidity in detecting target genes, we acknowledge the oversight in explicitly addressing this aspect in the manuscript. We agree that it is a crucial point, especially considering its inclusion in the title. We have included a statement clearly describing the turnaround time of the assays (68 minutes) in the materials and methods section. Furthermore, the rapidity of the assay was also clearly described in the discussion section of the manuscript (Line 362). This addition will ensure a comprehensive fulfilment of Objective 2 and provide a more robust analysis of our assay's performance.

We have also addressed the minor comments you provided:

- Line 32: Revised to "cefotaxime-supplemented agar plates."
- Lines 67-68: The accepted scientific notation for writing species names has been revised throughout the manuscript.
- Line 130: Since the AriaMx Real-Time PCR System is a fully integrated qPCR amplification, detection, and data analysis system, we did not see the need to include this description in the methods.
- Line 219: A reference supporting the assertion that blaSHV is intrinsic to K. pneumoniae was included in the manuscript (Line 408 - 410).
- Lines 336-338: This has been revised to eliminate redundancy and enhance clarity.
- Lines 419-420: We have addressed the incomplete sentence in the conclusion section.

Reviewer 4:

We appreciate your thorough review of our manuscript, your constructive comments are invaluable, and we are grateful for the insights you provided. Here are our responses to your specific comments:

- Cost comparison of multiplex qPCR vs Culture: We agree that providing a cost comparison between multiplex RT-PCR and culture methods in our African setting is a relevant aspect to address. However, our focus was limited to the rapidity and modification of the assay. In the future, we will consider the practicality and potential advantages of our multiplex RT-PCR approach, especially in regions where qPCR might face challenges.
- Italicization of Genus and Species Names in Supplementary Tables: We acknowledge the oversight in not italicizing genus and species names in S2 and 6 Tables. This has been rectified in the revised version of the manuscript.
- Representation of Common Genes and Human Isolates in States: We appreciate your suggestion for a more streamlined presentation of common genes across states, particularly in the context of human isolates. However, because of the limited number of isolates (*Enterobacter cloacae* and *Klebsiella pneumoniae*) this was not considered but we already have a manuscript published describing the variations of the AMR genes in Escherichia coli from the different states (Olorunleke SO, Kirchner M, Duggett N, AbuOun M, Okorie-Kanu OJ, Stevens K, et al. Molecular characterization of extended-spectrum cephalosporin-resistant Escherichia coli isolated from livestock and in-contact humans in Southeast Nigeria. Front Microbiol. 2022;0: 2801. doi: <https://10.3389/FMICB.2022.937968> )
- Incomplete Sentence and Recasting of Lines (420-423): This has been revised.
- Attribution of Highest Prevalence in Abia (Lines 387-390): We understand your point about the attribution of the highest prevalence of blaTEM and blaSHV in Abia to factors beyond its commercial hub status. We have added a study limitation section to the manuscript to acknowledge certain limitations about the sample size and the restricted number of *Klebsiella* and *Enterobacter spp* tested.

In conclusion, we believe that the revisions made in response to the reviewers' comments have significantly strengthened the manuscript. We are confident that the updated version addresses the concerns raised and improves the overall quality of the research.

We sincerely hope that the revisions meet the expectations of the reviewers and the editorial team. We are committed to further refining the manuscript if necessary and appreciate the opportunity to contribute to the scholarly discourse in PLOS ONE

Thank you for considering our manuscript, and we look forward to hearing from you soon.

Sincerely,

Solomon O. Olorunleke PhD

Department of Animal Science

Ebonyi State University, Abakaliki

[solomon.olorunleke@ebsu.edu.ng](mailto:solomon.olorunleke@ebsu.edu.ng)
