# Supplementary material for: Rapid detection and molecular epidemiology of β-lactamase producing Enterobacteriaceae isolated from food animals and in-contact humans in Nigeria
Source: PLoS One. 2024 Apr 11;19(4):e0289190. doi: 10.1371/journal.pone.0289190 (PMC11008865; doi:10.1371/journal.pone.0289190)
Supplement: Attachment — Submitted filename: Reviewer comment 1.docx [file pone.0289190.s007.docx]

This article presents a modified a multiplex real-time (RT)-PCR that can concurrently detect β-lactamase genes (blaCTX-M, blaTEM, and blaSHV) and Enterobacteriaceae 16S ribosomal RNA and provides useful data on the molecular epidemiology of AMR gene acquisition among Enterobacteriaceae

This study has prospect to be useful to the international AMR community, however there are some major deficiencies that needs to be addressed.

- The use of the acronym “RT-PCR” can be misleading as this is commonly used to represent reverse transcription PCR. Use of “qPCR” will be more appropriate.
- The study conclusion and recommendation seemed to be detached from the main study as the ESKAPE pathogens was not the main focus of this study, but Enterobacteriaceae of which only represent 3 out of the 6 ESKAPE pathogens. Moreover, the recommendation that ESKAPE pathogens be included in surveillance is strange as they are already identified as priority pathogens for AMR surveillance (at least in humans). I suggest conclusion be based on study findings and implication
- The interpretation of sensitivity and specificity should be done with caution considering the limited number of control strains and test isolate tested in this study. Especially the limited number of negative controls used in the control strain (4) and the test strains were selected based on their being able to grow in cefotaxime supplemented plates reducing the including of non ESBL producing strains
- The authors need to provide justification for focusing on the 3 ESBL genes with references to back it up.
- Figure 1 shows 22 control strains as opposed to 21 control strain which was indicated in the method.
- Table 1 adds little or no value to this article since all the reported parameters are 100%. It can be replaced with a sentence indicating 100% sensitivity, specificity, accuracy and precision.
- There is need to include well-articulated study limitations for example limited sample size, limited number of Klebsiella and Enterobacter tested etc
- The discussion in the current state seems to be quite repetitive of study findings. The authors should focus on discussing the key findings and their implication.

Other minor revisions include.

Pg 4 Line 85- “However, methods such as whole genome sequencing…….” This sentence is unwinding and needs to be rephrased.

Page 4 Line 74- I would use “affordable” rather than “cheap.”

Pg 5 Line 98: “evaluate the ability of this assay to rapidly detect these AMR genes in Enterobacteriaceae” I am a bit reserved about the use of “rapid”. PCR based test can hardly be regarded as rapid. Maybe when compared to traditional culture method but still constrained for point-of-care.

Pg 5 Line 106-107: Kindly provide more information on the control strains used in this section for example, how many positive and negative for ESBL of choice. More information about the source of control strains APHA should be provided in this section e.g. location

Pg 7 Line 151: “R gene”??? Do you mean resistant genes. Please provide initial definition

Pg 7 Line 159: Please write WGS in full since referring to “whole genome sequence” and not “whole genome sequencing.”

Pg 8 Line 168: “forward for inclusion any variable…”. Missing the word “of”

Pg 12 Line 227: The use of “as expected” is not necessary and should be expunged. Also quite repetitive across the manuscript

Pg 13 Table 3: Any reason why state level distribution was excluded from this table?

Pg 19 Line 336-346: This whole paragraph is a revision of literature and should be taken to the introduction section. The discussion section should focus on the implication of the study findings.

Pg 21 Line 383-386: “Although in this study, the state from which samples……” Take to study limitation.

Pg 22 Line 420: The sentence seems to be truncated.
